# Reduced graphene oxide aerogel with high-rate supercapacitive performance in aqueous electrolytes

**DOI:** 10.1186/1556-276X-8-247

**Published:** 2013-05-21

**Authors:** Weijiang Si, Xiaozhong Wu, Jin Zhou, Feifei Guo, Shuping Zhuo, Hongyou Cui, Wei Xing

**Affiliations:** 1 School of Chemical Engineering, Shandong University of Technology, Zibo 255049, People’s Republic of China; 2 School of Science, State Key Laboratory of Heavy Oil Processing, China University of Petroleum, Qingdao 266580, People’s Republic of China

**Keywords:** Supercapacitor, Reduced graphene oxide aerogel, Current density, Cyclic voltammetry

## Abstract

Reduced graphene oxide aerogel (RGOA) is synthesized successfully through a simultaneous self-assembly and reduction process using hypophosphorous acid and I_2_ as reductant. Nitrogen sorption analysis shows that the Brunauer-Emmett-Teller surface area of RGOA could reach as high as 830 m^2^ g^−1^, which is the largest value ever reported for graphene-based aerogels obtained through the simultaneous self-assembly and reduction strategy. The as-prepared RGOA is characterized by a variety of means such as scanning electron microscopy, transmission electron microscopy, X-ray diffraction, Raman spectroscopy, and X-ray photoelectron spectroscopy. Electrochemical tests show that RGOA exhibits a high-rate supercapacitive performance in aqueous electrolytes. The specific capacitance of RGOA is calculated to be 211.8 and 278.6 F g^−1^ in KOH and H_2_SO_4_ electrolytes, respectively. The perfect supercapacitive performance of RGOA is ascribed to its three-dimensional structure and the existence of oxygen-containing groups.

## Background

As a novel energy storage device that bridges the gap between conventional capacitors and batteries, supercapacitor has attracted much attention for its high power density and long cyclic life [1]. The studies about supercapacitor mainly focus on the electrode materials such as transition metal oxides, conducting polymers, and particularly carbon materials that are perfect electrode materials because of their good conductivity, cyclic stability, and large specific surface area [2-4]. Carbon materials with different structures such as carbon nanotubes, carbon nanofibers, hierarchical porous carbons, and ordered mesoporous carbons are widely studied in recent years [5-8]. Apart from these carbon materials, graphene and graphene-based materials have also been widely studied as electrode materials of supercapacitor [9-13]. Graphene is a two-dimensional sheet of *sp*^2^-hybridized carbon, which possesses many remarkable properties such as high surface area, excellent mechanical strength, and low electrical resistivity [14,15]. However, the practical preparation (chemical reduction process) of graphene-based material is often accompanied by the sacrifice of graphene surface area because the graphene layers are easy to restack through a *π*-*π* interaction during the chemical reduction process.

In order to obtain graphene-based material with high specific surface area, many researchers have prepared graphene-based materials with three-dimensional architecture. As a typical three-dimensional graphene-based material that has attracted much attention of researchers, graphene aerogel is often synthesized mainly through two strategies currently: self-assembly during reduction process [16-20] and post-reduction process after self-assembly [21-24]. Employing the first method, Xu et al. prepared graphene aerogel via self-assembly of graphene oxide during a hydrothermal reduction process at 180°C [16]. Chen synthesized graphene aerogel using various reductants such as NaHSO_3_, Na_2_S, vitamin C, and HI [17]. The specific surface area of the as-prepared graphene aerogels could only reach up to 512 m^2^ g^−1^[20] because the reduction of graphene oxide was accompanied by the elimination of oxygen-containing groups in aqueous solution. This could lead to the hydrophobility increase of reduced graphene oxide, thus resulting in the restacking of graphene sheets. Adopting the second method, we prepared the graphene aerogel with a superhigh C/O molar ratio by hydrogen reduction [21]. Worsley et al. synthesized a graphene aerogel through the self-assembly process in a basic solution followed by thermal reduction under nitrogen atmosphere. The Brunauer-Emmett-Teller (BET) surface area of the as-prepared graphene aerogel could reach as high as 1,300 m^2^ g^−1^, which is the largest value ever reported in the literatures [22]. Although the graphene aerogels possess large BET surface area when employing the second strategy, the preparation procedure is complex due to the separated self-assembly and reduction processes. It usually takes 72 h to finish the separate self-assembly process [23]. How to produce graphene aerogel with high surface area in a simple way is still a challenge currently.

Apart from the high surface area, the surface properties should also be taken into consideration while graphene-based material is used as electrode material in supercapacitor. The existence of surface functional groups is the characteristic surface properties of graphene-based materials made by Hummers' method. Graphene materials with functional surface often have a better dispersibility in aqueous electrolyte. Moreover, these functional groups may also generate pseudocapacitance in aqueous electrolytes. Xu's study indicates that graphene oxide is more suitable for supercapacitor application than graphene due to the existence of pseudocapacitance generated from the oxygen-containing groups [25]. Our previous work also shows that graphene oxide aerogel possesses a higher specific capacitance than graphene aerogel at low current densities in KOH electrolyte [21]. Thus, it would be promising to prepare high surface area graphene-based aerogels with functional surface for supercapacitor applications.

Herein, we synthesize a partially reduced graphene oxide aerogel (RGOA) through a simultaneous self-assembly and reduction process using hypophosphorous acid (HPA) and I_2_ as the reductants. Nitrogen sorption analysis shows that the specific surface area of the as-prepared RGOA could reach as high as 830 m^2^ g^−1^, which is the largest specific surface area ever reported for graphene aerogels obtained through the simultaneous self-assembly and reduction strategy. Electrochemical tests show that RGOA exhibits a high-rate supercapacitive performance in aqueous electrolytes. The specific capacitance of the RGOA can reach 211.8 and 278.6 F g^−1^ in KOH and H_2_SO_4_ electrolytes, respectively.

## Methods

### Material preparation

Graphite powder was purchased from Qingdao Ruisheng Graphite Co., Ltd. (Shandong, China). All other chemicals were purchased from Shanghai Chemical Reagents Company (Shanghai, China) and used directly without further purification. Graphite oxide was prepared according to Hummers' method [26]. Graphene oxide solution (5 mg mL^−1^) was acquired by dispersing graphite oxide in deionized water under ultrasonication. The reduced graphene oxide hydrogel was prepared according to Phams' method [18]. In a typical experiment, 5 g I_2_ was dissolved in 100 g HPA solution (50 wt.%), and then a 100-mL graphene oxide solution was added and sonicated for 5 min before transferred into an oven and aged at 90°C for 12 h. The obtained product was washed twice with acetone in a Soxhlet extractor (ISOPAD, Heidelberg, Germany) for 12 h to get reduced graphene oxide gels. The wet gels were dried with supercritical CO_2_ to obtain reduced graphene oxide aerogel, which was labeled as RGOA.

### Material characterization

The microstructure of the samples was characterized by X-ray diffraction (XRD, D8 Advance, Bruker Optik Gmbh, Ettlingen, Germany) and Raman spectroscopy (RM2000, Renishaw, Gloucestershire, UK). The thickness of graphite oxide sheet was examined using an atomic force microscope (AFM, Multimode NS3A, Veeco Instruments Inc., Plainview, NY, USA). The microscopic morphology of the samples was observed using a scanning electron microscope (SEM, FEI, Eindhoven, The Netherlands) and a transmission electron microscope (TEM, JEOL2010, Akishima, Tokyo, Japan). The surface properties of the samples were characterized by X-ray photoelectron spectroscopy (XPS, Escalab 250, Thermo VG Scientific, Waltham, MA, USA) and Fourier transform infrared spectroscopy (FT-IR, Nicolet 5700, Thermo Electron Corporation, Waltham, MA, USA). Nitrogen sorption measurement was performed with an ASAP 2020M analyzer (Micromeritics, Norcross, GA, USA) to obtain the specific surface area and pore structure parameters of the sample.

### Electrochemical measurements

Working electrodes were made by pressing RGOA onto the nickel foam and titanium mesh for 6 M KOH and 1 M H_2_SO_4_ electrolytes, respectively. The mass of active materials in each electrode was about 2 mg. In order to ensure that the electrode materials were thoroughly wetted with the electrolyte, the working electrodes were vacuum-impregnated with the electrolytes before electrochemical tests. The electrochemical capacitive performances of the sample were studied on a CHI660D electrochemical workstation. Electrochemical measurements including cyclic voltammetry (CV), galvanostatic charge–discharge, and electrochemical impedance spectroscopy (EIS) were performed in a three-electrode system using a platinum film as a counter electrode and a saturated calomel electrode (SCE) as a reference electrode. Potential windows of −1 ~ 0 V and 0 ~ 1 V vs. SCE reference electrode were applied to the electrochemical measurements in KOH and H_2_SO_4_ electrolytes, respectively. In addition, the electrochemical performance of RGOA was also evaluated using a two-electrode system in H_2_SO_4_ electrolyte with a potential window of 0 ~ 1.2 V.

## Results and discussion

### Morphological evolution

**Figure 1:**
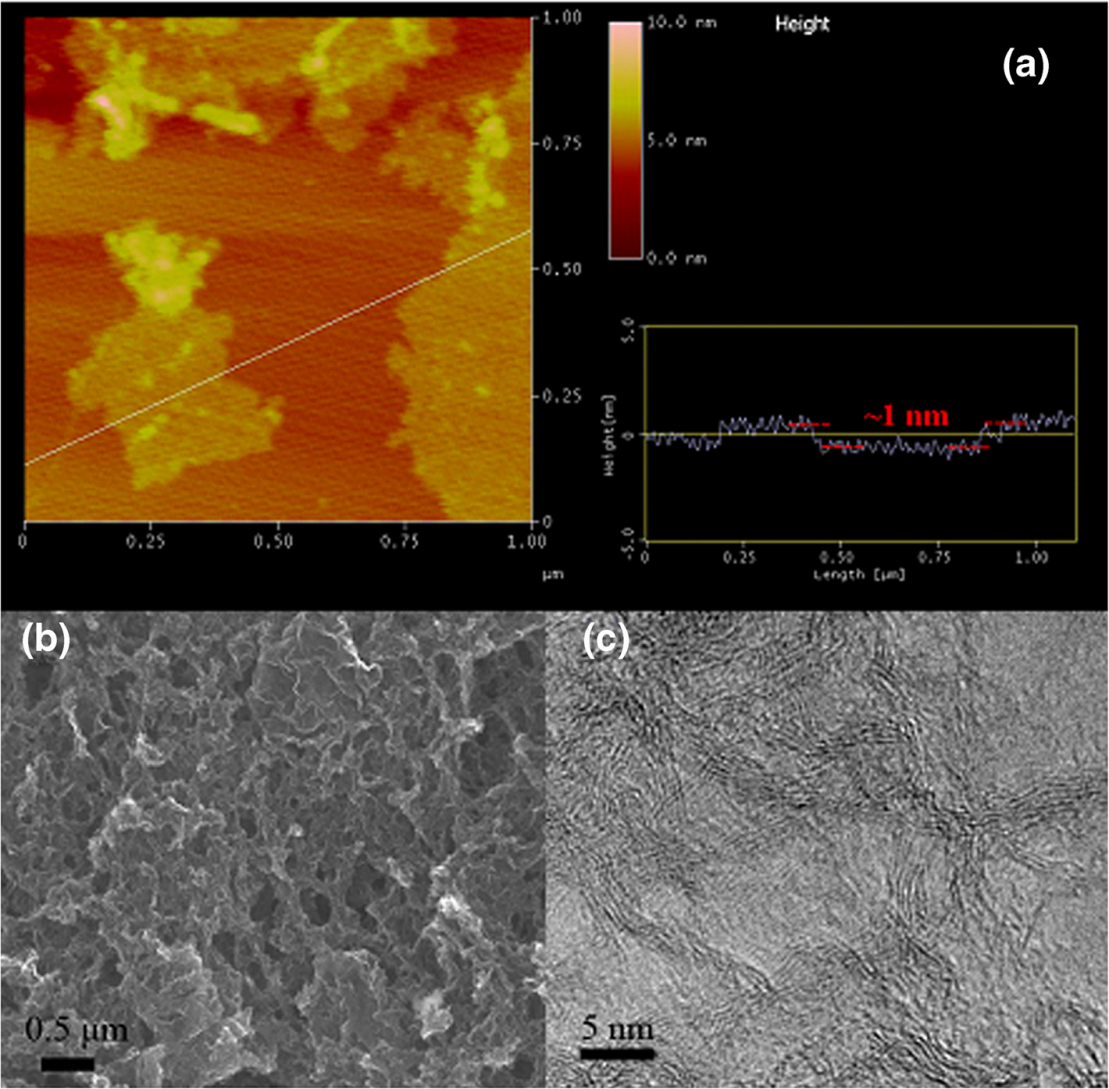
**Microstructural observations for samples.** (**a**) AFM image of graphite oxide sheets with height profile. (**b**) SEM and (**c**) TEM images of RGOA.

AFM image of graphite oxide (GO) (Figure 1a) shows that the size of prepared GO sheets is in a range of several hundred nanometers to 1 μm, and the AFM height profile of GO sheets reveals that the obtained GO sheets are monolayered (approximately 1 nm). SEM image (Figure 1b) indicates that RGOA is composed of randomly oriented GO/graphene sheets, forming a three-dimensional structure. Plentiful mesopores and macropores are found in the bulk of RGOA, suggesting the formation of a porous material. TEM image reveals that RGOA presents an ordered graphitic structure with curved graphene sheets. The formation of graphitic structure indicates a high reduction degree of graphene oxide during the preparation process.

### Structural evolution

**Figure 2:**
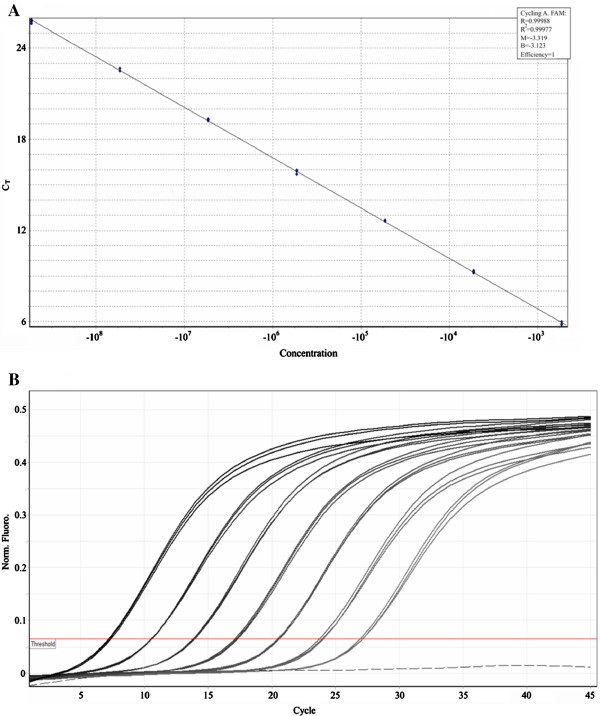
**Structural analyses for samples.** (**a**) N_2_ sorption isotherm and (**b**) pore size distribution curve of RGOA. (**c**) XRD patterns and (**d**) Raman spectra of GO and RGOA.

Type IV adsorption isotherm is observed for RGOA (Figure 2a), indicating that the aerogel is a mesoporous material. The obvious hysteresis loop can be observed at relative pressures ranging from 0.42 to 1.0. The pore size distribution curve (Figure 2b) derived from desorption branch by the Barret-Joyner-Halenda method shows that most of the pores distribute within a range of 2 to 50 nm with a most probable pore diameter of approximately 4 nm. The BET specific surface area is calculated to be 830 m^2^ g^−1^, which is the largest value ever reported for graphene-based aerogel materials prepared by a simultaneous self-assembly and reduction method. The interlayer distance of GO calculated from the (002) peak in XRD pattern (Figure 2C) is 0.71 nm, which is much larger than that of pristine graphite (approximately 0.34 nm) owing to the fact that plenty of oxygen-containing groups, such as hydroxyl, epoxyl, and carboxyl, are introduced onto graphene layers during the oxidation process. Compared with GO, the XRD pattern of RGOA exhibits a broad diffraction peak at 2*θ* = 24° corresponding to the (002) plane of graphite structure. The formation of graphite-like structure of RGOA indicates the efficient removal of oxygen-containing groups from GO during the simultaneous self-assembly and reduction process. For the purpose of exploring the structural and electronic properties, including disordered and defect structures, of RGOA, Raman spectroscopy analyses are also conducted (Figure 2d). There are two prominent peaks at approximately 1,355 and approximately 1,600 cm^−1^ corresponding to the D and G band, respectively. It has been reported that the D band originates from the disorder-induced mode associated with structural defects and imperfections, while the G band corresponds to the first-order scattering of the *E*_2g_ mode from the *sp*^2^ carbon domains [27]. The intensity ratio *I*_D_/*I*_G_ is often used as a measure of the disorder in graphitic materials [28]. The increased *I*_D_/*I*_G_ value indicates the restoration of *sp*^2^ C=C bonds in graphitic structure when oxygen-containing groups escape from GO. Moreover, the decrease of full-width at half maximum of G band indicates a high graphitization degree of RGOA as well [29,30]. These results coincide well with what was reflected from XRD analyses and TEM observations.

### Evolution of surface properties

**Figure 3:**
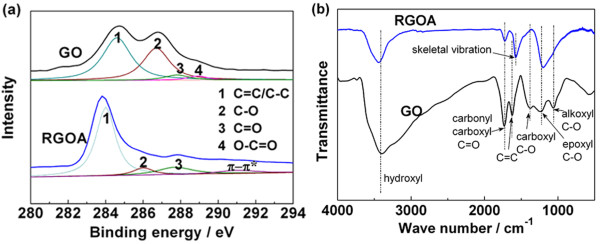
**C1*s *XPS spectra (a) and FT-IR spectra (b) of GO and RGOA.**

XPS analyses are conducted for GO and RGOA (Figure 3a) to investigate the changes of surface oxygen-containing species during the preparation process. The C1*s* spectrum of GO can be deconvoluted into four peaks at 284.6, 286.7, 287.8, and 289 eV, corresponding to C=C/C-C in aromatic rings, C-O in alkoxyl and epoxyl, C=O in carbonyl, and O-C=O in carboxyl groups, respectively [30-33]. When GO is reduced, the peak intensity of C=C/C-C in aromatic rings rises dramatically, while those of C-O and C=O decrease sharply, and the peak of O-C=O disappears, clearly suggesting the efficient removal of oxygen-containing groups and the restoration of C=C/C-C structure in graphitic structure. It should also be noted that a new peak emerges at 291 eV corresponding to the *π*-π* shake-up satellite peak, indicating that the delocalized *π* conjugation is restored [34,35]. C/O molar ratios calculated according to the XPS analyses are 2.3 and 6.1 for GO and RGOA, respectively. FT-IR is also adopted to analyze the evolution of oxygen-containing groups during the self-assembly and reduction process (Figure 3b). As for GO, the following characteristic peaks are observed: O-H stretching vibrations (3,000 ~ 3,500 cm^−1^), C=O stretching vibrations from carbonyl and carboxyl groups (approximately 1,720 cm^−1^), C=C stretching or skeletal vibrations from unoxidized graphitic domains (approximately 1,620 cm^−1^), O-H bending vibrations from hydroxyl groups (approximately 1,400 cm^−1^), C-O stretching vibration from epoxyl (approximately 1,226 cm^−1^), and alkoxyl (approximately 1,052 cm^−1^) [27,36]. There is a dramatic decrease of hydroxyl, C-O and C=O groups after the reduction process. A new featured peak at 1,568 cm^−1^ due to the skeletal vibration of graphene sheets appears. Combining the results of XPS and FT-IR analyses, partial oxygen-containing groups are still retained after the self-assembly and reduction process although there is a significant decrease of such functional groups.

### Electrochemical capacitive performances

#### Three-electrode system

**Figure 4:**
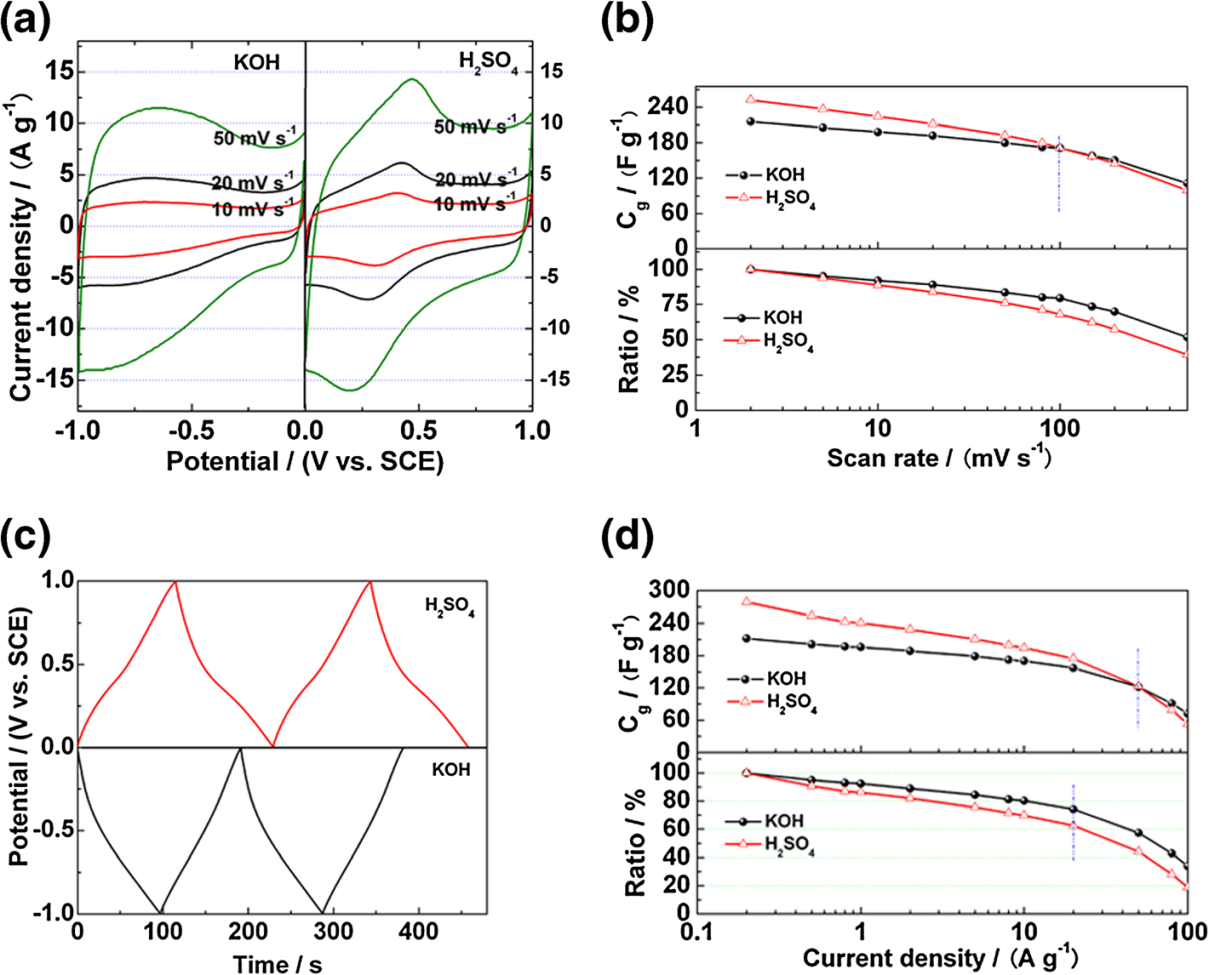
**Electrochemical performance of RGOA in KOH and H**_**2**_**SO**_**4 **_**electrolytes.** (**a**) Cyclic voltammograms at the voltage scan rates of 10, 20, and 50 mV s^−1^. (**b**) Plots of specific capacitance and its retention ratio vs. voltage scan rate. (**c**) Galvanostatic charge–discharge curves at a current density of 2 A g^−1^. (**d**) Plots of specific capacitance and its retention ratio vs. current density.

Cyclic voltammograms of RGOA at different scan rates in KOH and H_2_SO_4_ are shown in Figure 4a. The CV curves in both electrolytes show a rectangular-like shape, which is attributed to the electric double-layer capacitance in each potential window. As for the CV curves in KOH electrolyte, although there is no obvious redox peaks, RGOA also exhibits pseudocapacitance besides electric double-layer capacitance at the potential window of −1.0 ~ −0.3 V because the current density severely changes as the potential varies within this potential window [21]. An equilibrium redox reaction probably occurs as follows within this potential window [37]:

$$-C_{X}O+e^{-}+K^{+}\Leftrightarrow -C_{X}-\mathrm{OK}.$$

contrast, there are obvious redox peaks within the potential window of 0.0 ~ 0.6 V in H_2_SO_4_ electrolyte, which are thought to be derived from the following redox reactions [38,39]:

$$>C-\mathrm{OH}\Leftrightarrow C=O+H^{+}+e^{-},$$

In addition, the current density at each scan rate in H_2_SO_4_ electrolyte is higher than that in KOH electrolyte, which indicates that oxygen-containing groups exhibit more pseudocapacitance in acid electrolyte. Therefore, as shown in Figure 4b, the specific capacitance calculated from CV curves displays that RGOA possesses larger capacitance in H_2_SO_4_ electrolyte when the scan rates are lower than 100 mV s^−1^. However, RGOA maintains a higher capacitance in KOH electrolyte when the scan rates exceed 100 mV s^−1^, which is probably due to the higher ionic concentration of KOH electrolyte than that of H_2_SO_4_ electrolyte. The galvanostatic charge–discharge curves of RGOA in different electrolytes are composed of two parts: the first part is within the potential window of 0.0 ~ −0.3 V in KOH electrolyte and 0.6 ~ 1.0 V in H_2_SO_4_ electrolyte, which is attributed to the electric double-layer capacitance. The other part exhibits a longer duration time, indicating the existence of pseudocapacitance besides the electric double-layer capacitance. As shown in Figure 4d, capacitance retention ratios of RGOA remain 74% and 63% in KOH and H_2_SO_4_ electrolytes when current density increases from 0.2 to 20 A g^−1^, exhibiting a high-rate capacitive performance. This high-rate performance is mainly attributed to the three-dimensional structure, which is beneficial for the ionic diffusion of electrolyte to the inner pores of bulk material. As shown in Figure 4d, the specific capacitances are calculated to be 211.8 and 278.6 F g^−1^ in KOH and H_2_SO_4_ electrolytes at the current density of 0.2 A g^−1^. The specific capacitances per surface area are calculated to be 25.5 and 33.6 μF cm^−2^ in KOH and H_2_SO_4_ electrolytes, respectively, indicating more pseudocapacitance in H_2_SO_4_ electrolyte. These results coincide well with the cyclic voltammetry measurements.

**Figure 5:**
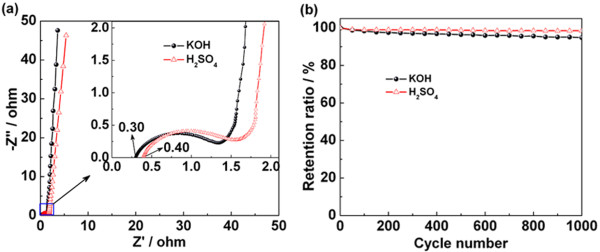
**Nyquist plot (a) and cycle tests (b) in electrolytes of KOH and H**_**2**_**SO**_**4**_**.**

EIS is adopted to investigate the chemical and physical processes occurring on the electrode surface. The Nyquist plots of RGOA in different electrolytes are shown in Figure 5a. Within the low-frequency region, the curve in KOH electrolyte is more parallel to the ordinate than that in H_2_SO_4_ electrolyte, indicating a better capacitive behavior in KOH electrolyte. The intersection of the curve with the abscissa represents equivalent series resistance [40]. This value is due to the combination of the following: (a) ionic and electronic charge-transfer resistances, (b) intrinsic charge-transfer resistance of the active material, and (c) diffusive as well as contact resistance at the active material/current collector interface [41]. It can be seen from the inset in Figure 5a that these resistance values are 0.30 and 0.40 Ω for KOH and H_2_SO_4_ electrolytes, respectively. This is mainly attributed to the different ionic concentration of electrolytes. The semicircular loop at high frequencies is due to the charge transfer resistance of the electrode, which is attributed to the faradaic redox process in the system. The charge-transfer resistances *R*_ct_ can be estimated from the diameter of this semicircle to be 1.03 and 1.16 Ω in KOH and H_2_SO_4_ electrolytes, respectively, which indicates a more pseudocapacitance in H_2_SO_4_. This result coincides well with the results from cyclic voltammetry and galvanostatic charge–discharge measurements. Figure 5b shows the cycle stability of RGOA through cyclic voltammetry measurements. The capacitance retention ratio reaches 98.5% after 1,000 cycles in H_2_SO_4_, which is larger than that in KOH electrolyte.

### Two-electrode system

**Figure 6:**
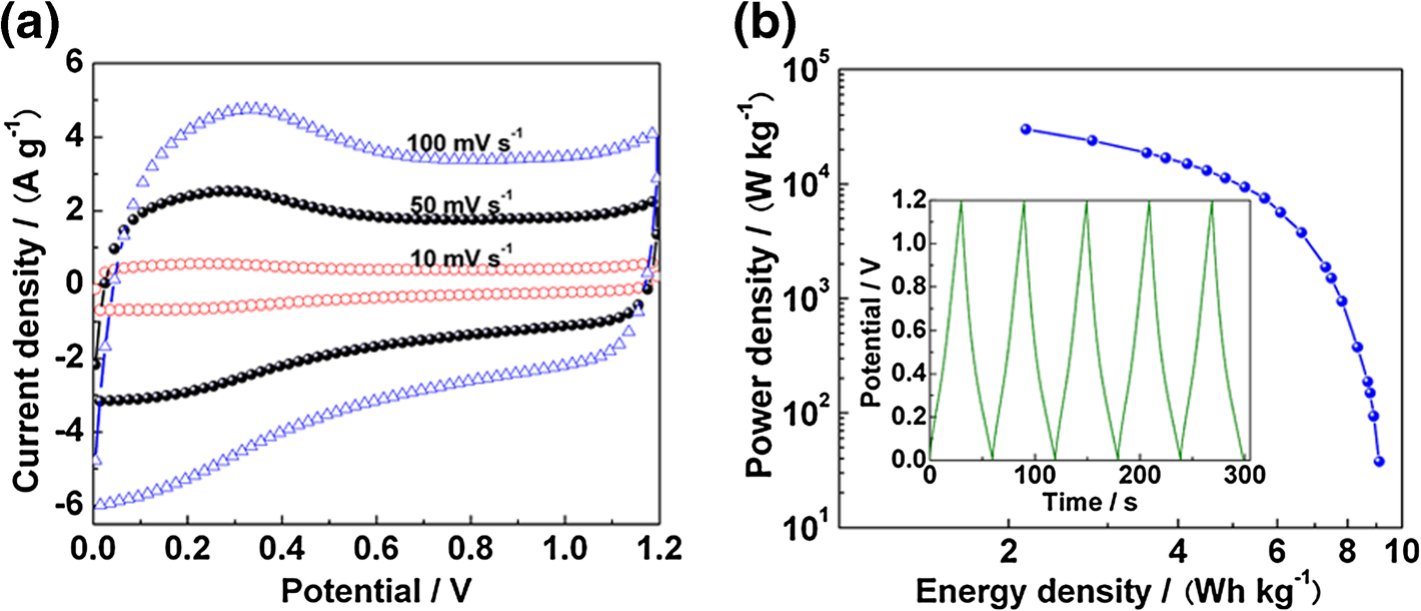
**Supercapacitive performance of RGOA in a two-electrode system.** (**a**) Cyclic voltammogramms at different scan rates. (**b**) Ragone plot and galvanostatic charge–discharge curves at a current density of 5 A g^−1^ (inset).

Considering the high specific capacitance and perfect cycle stability in H_2_SO_4_ electrolyte, RGOA electrodes are assembled into a supercapacitor cell and tested in a two-electrode system with a potential window of 0.0 ~ 1.2 V. The energy density (*E*) and power density (*P*) are calculated using Equations 1 and 2 [42]:

(1) $$E=\frac{1}{2}C_{\mathrm{cell}}V^{2},$$

(2) $$P=\frac{E}{\mathrm{\Delta t}},$$

where *C*_cell_ is the specific capacitance of the total cell, *V* is the cell potential, and Δ*t* is the discharge time. As shown in Figure 6a, the cyclic voltammogramms of RGOA basically show a rectangular shape even at high scan rates although there are obvious redox peaks, which indicates a combination of electric double-layer and pseudocapacitive capacitance formation mechanism. The galvanostatic charge–discharge curve (the inset in Figure 6b) shows a fine symmetry, indicating a perfect coulombic efficiency for supercapacitor cell. The Ragone plot in Figure 5b displays that RGOA exhibits a high energy density even at a large power density, which is superior to other graphene-based materials [43].

## Conclusions

A simultaneous self-assembly and reduction method is adopted to successfully synthesize the reduced graphene oxide aerogel with the specific surface area of 830 m^2^ g^−1^, which is the largest value ever reported for graphene-based aerogels obtained through the simultaneous self-assembly and reduction strategy. Systematic characterizations suggest that the as-prepared RGOA is a three-dimensional mesoporous material with functionalized surface. Electrochemical tests show that RGOA exhibits high-rate supercapacitive performance. Its specific capacitances reach as high as 211.8 and 278.6 F g^−1^ in KOH and H_2_SO_4_ electrolytes, respectively. The perfect supercapacitive performance of RGOA is ascribed to its three-dimensional structure and the existence of oxygen-containing groups.

## Competing interests

The authors declare that they have no competing interests.

## Authors’ contributions

WS and XW performed the experiments and drafted the manuscript together. JZ checked the figures and gave the final approval of the version to be published. FG performed partial experiments. SZ supervised the project. HC guided the experiment on the CO_2_ supercritical drying process of RGOA. WX guided the idea, revised, and finalized the manuscript. All authors read and approved the final manuscript.
